# First person – Martín Baccino-Calace

**DOI:** 10.1242/bio.055483

**Published:** 2020-08-18

## Abstract

First Person is a series of interviews with the first authors of a selection of papers published in Biology Open, helping early-career researchers promote themselves alongside their papers. Martín Baccino-Calace is first author on ‘Compartment and cell-type specific hypoxia responses in the developing *Drosophila* brain’, published in BiO. Martín conducted the research described in this article while a master's student in Rafael Cantera's lab at the Department of Neurodevelopment Biology, IIBCE, Uruguay. He is now a graduate student in the lab of Martin Müller at the Department of Molecular Life Sciences, University of Zurich, Switzerland, investigating synaptic physiology.


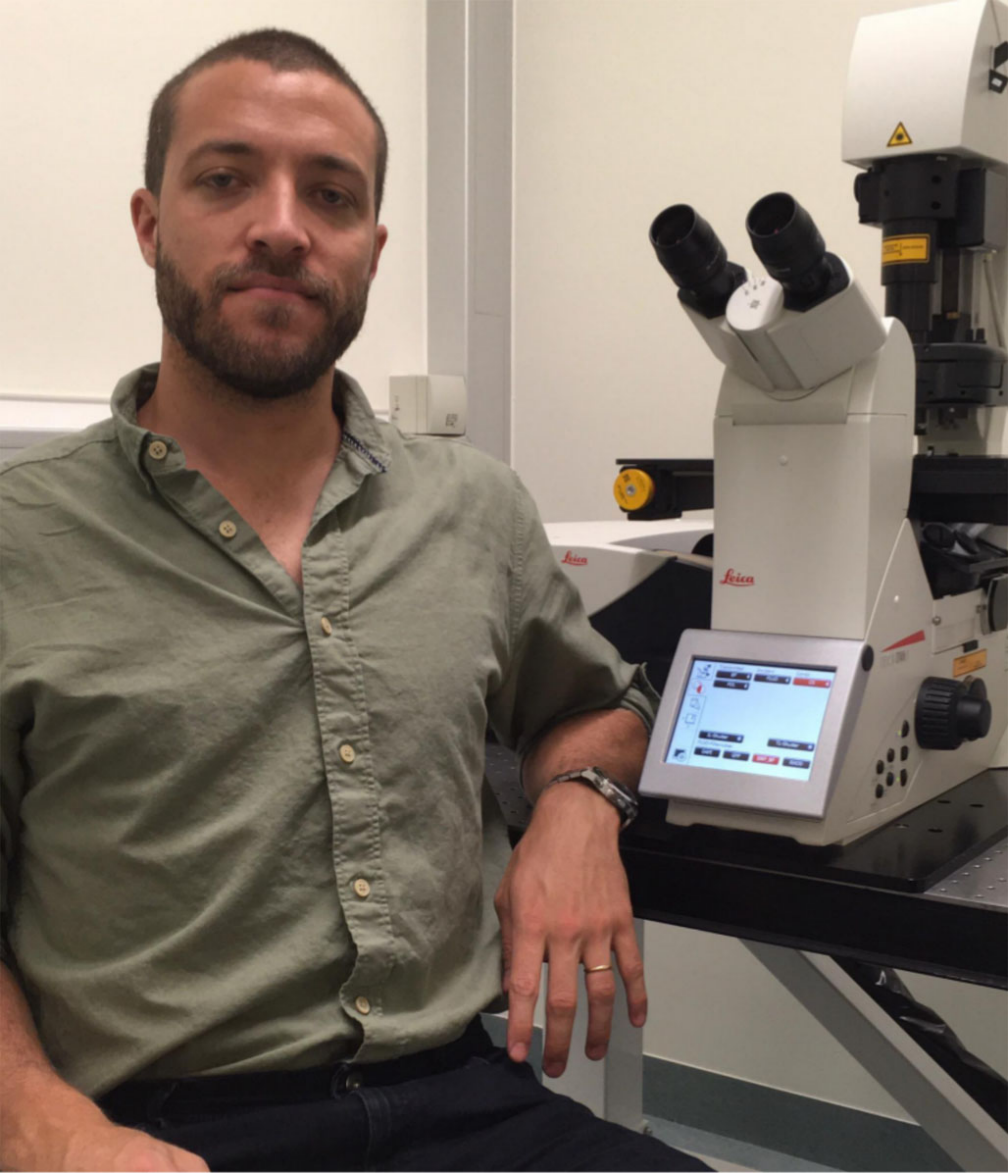


**Martín Baccino-Calace**

**What is your scientific background and the general focus of your lab?**

Towards the end of my bachelor's in biology, I was completely convinced that I wanted to start my research in the field of neuroscience. With this in mind, I joined the lab of Rafael Cantera, in Montevideo, Uruguay, which focuses on a wide range of different aspects of nervous system development. My experience in the lab was excellent in every aspect and is the reason why, after finishing my bachelor's degree, I started my master's project in the same lab. We worked on understanding the role of hypoxia on nervous system development using *Drosophila* as a model. During this project, I did two internships under the supervision of Boris Egger at the Department of Biology of the University of Fribourg, in Switzerland. There I was able to further my knowledge a great deal about confocal microscopy, *Drosophila* genetics, immunohistochemistry and some principles of molecular biology. These internships turned out to be life changing, both from a personal and scientific point of view, and would greatly affect the course of my academic career. During my stay, we initiated a collaboration with Stefan Luschnig and his PhD student Tvisha Misra, who at the time were both at the University of Zürich. They had just developed a genetically encoded hypoxia biosensor in *Drosophila*. We analyzed and validated the behavior of the sensor in the *Drosophila* brain and with help of Felix Meyenhofer designed an imaging analysis pipeline. Our project resulted in two manuscripts published in Biology Open (Misra et al., 2016; and now Baccino-Calace et al., 2020). After my master's I decided to move to Europe for my PhD thesis work. One event led to another and today I am in the middle of my PhD work in the lab of Martin Muller in Zürich. My goal is to uncover new molecular players in the process of presynaptic homeostatic plasticity.

**How would you explain the main findings of your paper to non-scientific family and friends?**

It is commonly said that oxygen is a double-edged sword. With it, organisms can break down glucose all the way to carbon dioxide in a way that yields approximately 16 times the energy output than the anaerobic alternative. At the same time, oxygen, a very reactive molecule, causes a lot of damage to macromolecules like proteins and DNA, which threatens the survival of cells. Stem cells are undifferentiated cells of multicellular organisms. They are capable of giving rise to more cells of the same type, from which other kinds of cell arise by differentiation, such as neurons and muscle cells. Many types of stem cells have been found to exist in regions where oxygen is relatively low, and it is hypothesized that this is an evolutionarily selected mechanism to protect and ensure the survival of these extremely important cells. In our work, we used a fluorescent oxygen sensor to show that a group of stem cells in the *Drosophila* larval brain reside in a region with lower oxygen relative to the rest of the brain. Moreover, we show that the distance from a cell to tracheoles (the air tubes used for respiration in insects and other animals), which carry oxygen to cells, is a good predictor of the level of oxygen of the cell. Furthermore, we show that the degree to which a cell has advanced in the process of differentiation correlates with its level of oxygenation. Our results suggest a mechanism by which stem cells are secluded in a low oxygen compartment during development, which protects them from oxygen-associated damage.

**What are the potential implications of these results for your field of research?**

We provide a very in-depth and quantitative description of hypoxia responses of an entire organ at an unprecedented cellular resolution. We think our work will serve as a basis for future research studying effects of hypoxia during normal brain development as well as in pathological situations such as tumorigenesis.

“We think our work will serve as a basis for future research studying effects of hypoxia during normal brain development as well as in pathological situations such as tumorigenesis.”

**What has surprised you the most while conducting your research?**

We initially discovered that the distance between a cell and the next closest tracheole is a good predictor of the hypoxic state of that cell. In this context, it was striking to find that different cell types differ in their hypoxia response although residing at seemingly equal distances to oxygen-carrying tracheoles. As an example, we observed that neuroblasts (a type of stem cell in the brain) were especially responsive to hypoxia. On the other hand, neuroepithelial cells (another type of stem cell) show virtually no hypoxia response, regardless of their distance to tracheoles. We conclude that oxygen availability is the major factor controlling the hypoxia response, but cell-intrinsic and cell-specific factors modulate this response in an unexpected manner.

**What, in your opinion, are some of the greatest achievements in your field and how has this influenced your research?**

Some of the most influential work in our field are the discoveries that led to The Nobel Prize in Physiology or Medicine for 2019 awarded to William Kaelin, Jr, Sir Peter Ratcliffe, and Gregg Semenza. Their combined work uncovered the molecular mechanisms through which cells sense and adapt to changes in oxygen availability. They showed that when oxygen is low, the HIF transcription factor is stabilized, which results in changes in gene expression that allow the cell to adapt to the level of available oxygen. Without this groundbreaking discovery, the HIF-based hypoxia sensor that we use in our publications would not be conceivable.

**Figure:**
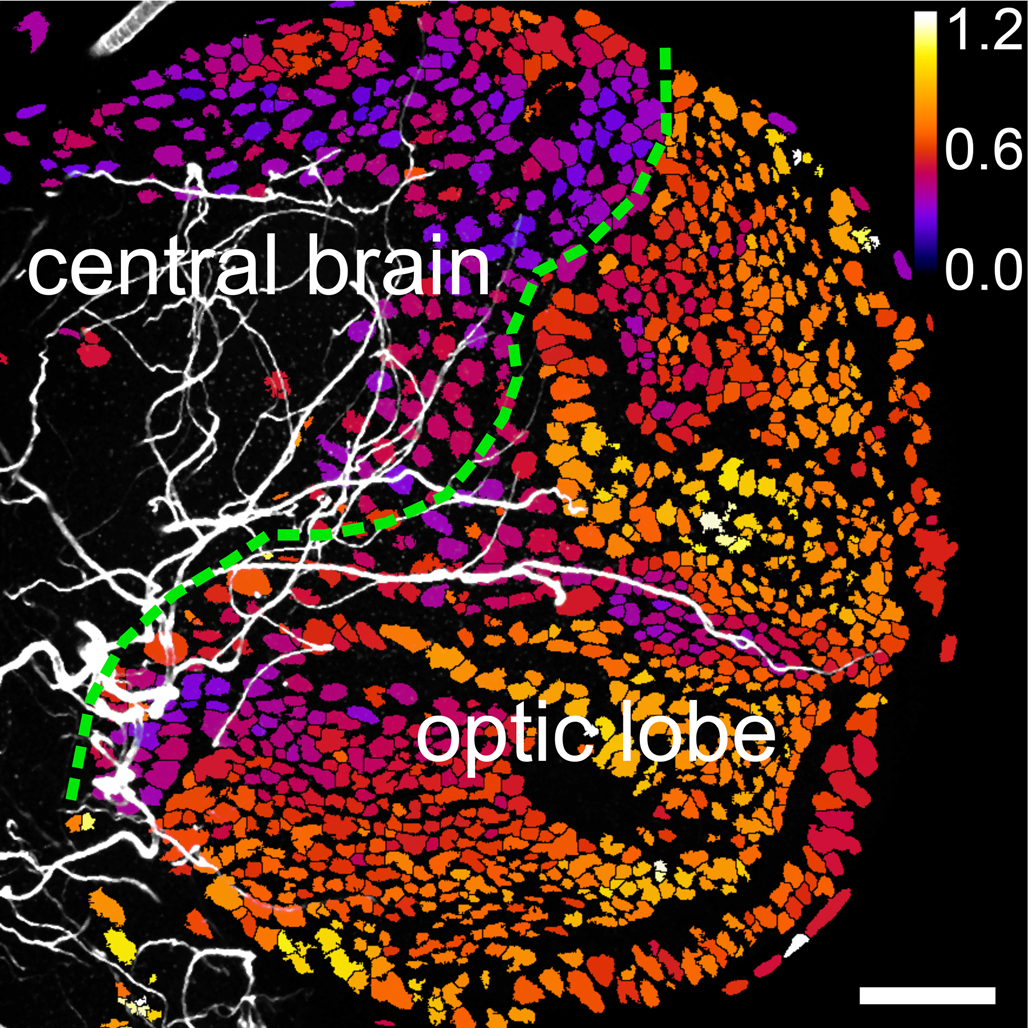
**A ratiometric image of a *Drosophila* larval brain obtained with a GFP/RFP-based genetically encoded oxygen sensor.** The central brain, where mostly differentiated functional cells reside, shows a dense network of tracheoles (air tubes; white), and consequentially, low levels of hypoxia response (well oxygenated, represented by dark colours). On the contrary, the optic lobe anlagen, which harbours the neural stem cells that will give rise to the adult optic lobe, contains much fewer tracheoles, which results in a high hypoxia response (indicating low oxygen availability, represented by bright colours). Scale bar: 40 μm.

**What changes do you think could improve the professional lives of early-career scientists?**

Regarding the current state of academia, I agree with Dr Romain Brette in that today we are facing a problem of academic precarity. Most of the work is done by graduate students or post-docs, positions that experience turn over in a matter of years, and who work under such a lot of pressure that it sometimes might even compromise the quality of research. Moreover, the number of students largely outnumbers the amount of available permanent positions, which in most countries consists only of the Principal Investigator (PI) position. I think that alternatives to the ‘one PI-one lab’ model could be of some help. Perhaps one alternative could be the creation of intermediate long-term research positions that don't involve running a lab.

**What's next for you?**

The present work published in Biology Open was done in the context of my master's project. After that, I moved to Zurich to work in the lab of Martin Muller (University of Zurich). At this moment, I am in the last year of my PhD in which I focused on uncovering the molecular players that control presynaptic homeostatic plasticity. At the end of my PhD, I will either move on to do a post-doc or transition into industry.
